# Novel Use of Silicone Sheets for Immediate Implant Placement in Fresh Molar Extraction Sockets

**DOI:** 10.1155/2019/3501671

**Published:** 2019-02-28

**Authors:** Won-Pyo Lee, Jae-Heung Choi, Sang-Joun Yu, Byung-Ock Kim

**Affiliations:** ^1^Department of Periodontology, School of Dentistry, Chosun University, 309 Pilmun-daero, Dong-gu, Gwangju 61452, Republic of Korea; ^2^Department of Preventive and Public Health Dentistry, School of Dentistry, Chosun University, 309 Pilmun-daero, Dong-gu, Gwangju 61452, Republic of Korea

## Abstract

In the recent years, the immediate placement of dental implants into fresh extraction sites has become an acceptable treatment approach. However, immediate molar implant placement presents specific challenges because of the anatomical and physiologic limitations. Such implant surgeries commonly require procedures that use a barrier membrane to generate bone and soft tissue or one that seals the molar extraction socket through a coronally advanced flap. Here, as an alternative, we report a method for treating molar extraction socket wounds in the hard and soft tissues after immediate placement of an implant using a silicone sheet.

## 1. Introduction

In the recent years, the immediate placement of an implant into the fresh extraction socket has become a popular surgical procedure, as it improves patient comport and shortens treatment period. This not only reduces the time until the implant can be restored but also results in fewer surgical procedures and has a high success rate [1–3].

However, because of anatomical and physiological limitations, the immediate placement of dental implants into fresh molar extraction sockets may be adversely affected by the osseous socket anatomy, lack of soft tissue closure, and flap dehiscence over the extraction site [4]. Such an implant surgery commonly requires procedures that use bone graft material and a barrier membrane to generate bone and soft tissue between the fixture and extraction socket [5] or one that seals the extraction socket through a coronally advanced flap [6]. However, these procedures require vast clinical experience, and the use of a barrier membrane is accompanied by an increase in the cost and risk of infection [7, 8]. Furthermore, additional flap manipulation involves a time-consuming series of procedures that causes unnecessary stress to patients [9, 10].

Medical grade silicone sheets have been widely used in the medical field. Because silicone sheets are available in a wide range of sizes and thicknesses, these sheets can be customized by the surgeon into different configurations and sizes to meet individual procedure requirements to provide flexibility for each patient [11–13].

Therefore, we hereby report a method for treating molar extraction socket wounds in hard and soft tissues after immediate placement of an implant using medical grade U-shaped silicone sheets.

## 2. Case Reports

### 2.1. Silicone Cap

**Figure 1:**
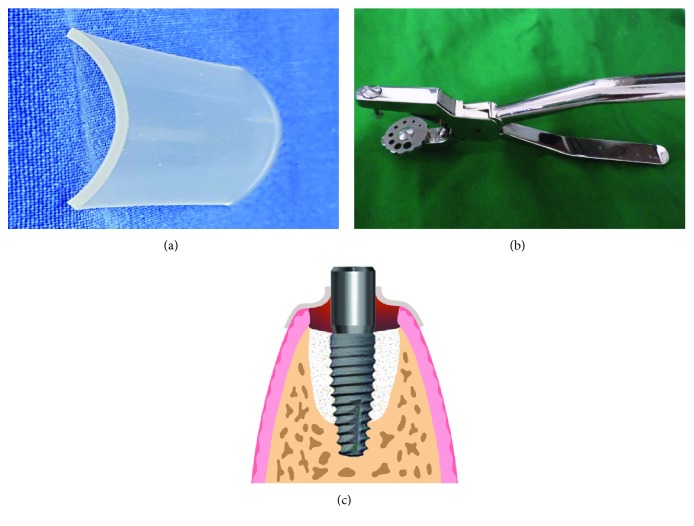
(a) Medical grade U-shaped silicone sheet. (b) Punching tool to produce silicone caps. (c) A schematic diagram of a silicone cap placed over a cylinder-type healing abutment.

The silicone cap was a custom-made silicone fragment. We used a commercially available, elastic, sterile, and U-shaped medical grade 1 mm thick silicone sheet (Kuwotech, Gwangju, Korea) (Figure 1(a)). Using an Irish scissor, we cut the silicone sheet at least 2–3 mm longer than the extraction socket in both the buccal and lingual directions, covering the extraction socket completely. We then made a hole with a diameter smaller than that of the healing abutment using a custom-made punching tool (Kuwotech, Gwangju, Korea) (Figure 1(b)). The diameter of the cylinder-type healing abutment (Kuwotech, Gwangju, Korea) was 4.5 mm and that of the hole in the silicone cap was 2.5 mm. When the silicone cap, stretched using pincettes, is placed over the healing abutment fastened to an implant fixture, the hole portion of the silicone cap that touches the healing abutment is pulled upward by frictional force. In response, a compressive force is generated in the soft tissue around the extraction socket at the margin of the U-shaped silicone cap (Figure 1(c)) [14].

### 2.2. Case 1

**Figure 2:**
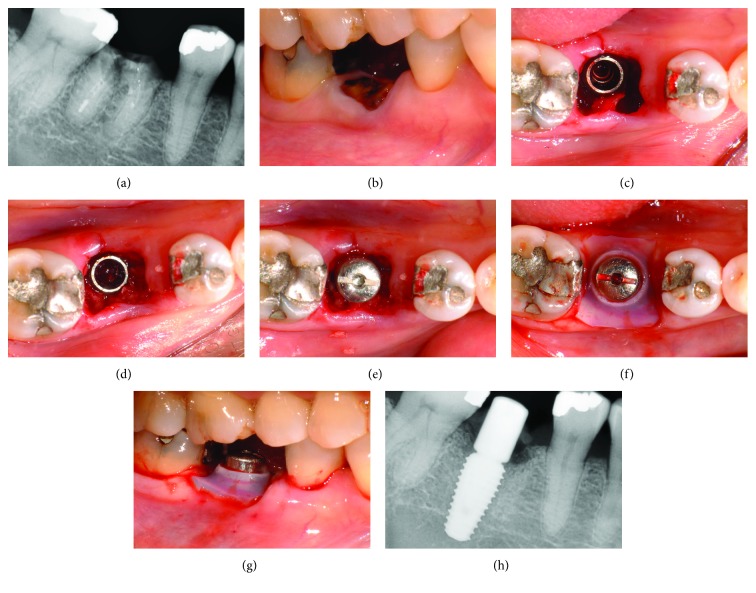
(a) Periapical radiograph of tooth 46 from the initial examination. (b) Buccal view of tooth 46 at the initial examination. (c) Implant inserted into interradicular septum. (d) Clinical condition after gap filling with autologous particulate bone. (e) Clinical condition after the installation of a cylinder-type healing abutment. (f) Occlusal view after the application of silicone cap on healing abutment. (g) Buccal view after the application of silicone cap on healing abutment. (h) Periapical radiograph immediately after surgery.

A 56-year-old female patient with an overall healthy oral state visited the clinic for implant placement after the removal of a retained right mandibular 1^st^ molar (tooth 46) root (Figures 2(a) and 2(b)). After local anesthesia, the dental root was separated using a high-speed handpiece and #558 bur, followed by careful atraumatic extraction using an elevator. After drilling into the interradicular septum using only a 4 mm diameter trephine bur to obtain autologous bone, a 10 mm long and 5 mm diameter implant (AnyOne, Megagen Implant Co., Seoul, Korea) was inserted 1 mm deeper than the height of the buccolingual alveolar ridge. The gap between the inserted fixture and extraction socket was about 4 mm in both the buccolingual and mesiodistal directions (Figure 2(c)). The gap between the extraction socket and implant was filled with autologous particulate bone obtained by grinding the bone core made by the trephine bur (Figure 2(d)). After equipping it with a cylinder-type healing abutment (Figure 2(e)), a silicone cap was fabricated as described earlier and then placed over the healing abutment because it was easily stretched using the pincettes (Figure 2(f)). Except for the force that exerted light pressure on the soft tissues around the extraction socket, the installed silicone cap showed no movement (Figure 2(g)). Thus, without making an incision or using sutures and a barrier membrane, the extraction socket was sealed using only autologous bone and a silicone cap. The silicone cap is seen as a radiopacity on a radiograph (Figure 2(h)).

**Figure 3:**
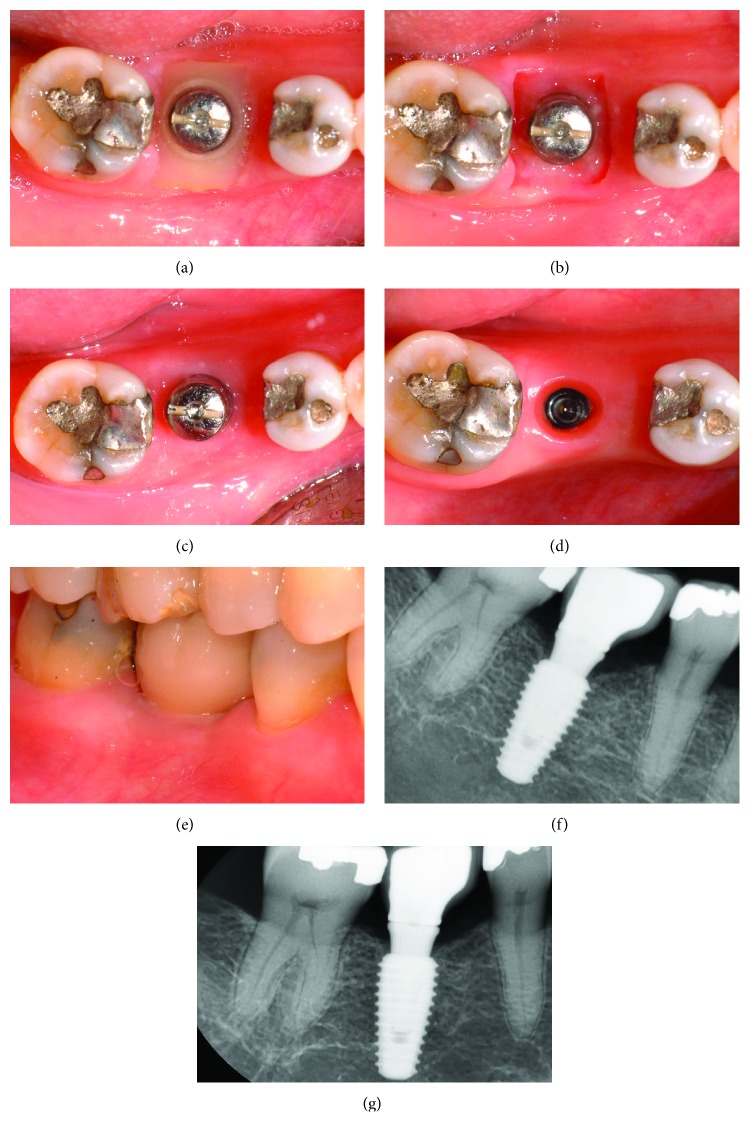
(a) Clinical condition before the removal of the silicone cap at 2 weeks postoperatively. (b) Soft tissue condition after the removal of the silicone cap at 2 weeks postoperatively. (c) Clinical condition at 2.5 months postoperatively. (d) Clinical condition at 6 months postoperatively before the application of the final prosthesis. (e) Buccal view at 6 months postoperatively with the final prosthesis. (f) Radiograph at 6 months postoperatively. (g) Radiograph at 22 months postoperatively.

The patient was instructed to be careful not to dislodge the cap with their tongue, masticatory movements, or by brushing. The patient was advised to rinse with a 0.1% chlorhexidine digluconate solution (Hexomedine solution, Bukwang Pharm. Co., Seoul, Korea) twice daily for 2 weeks. Additionally, the patient was prescribed with antibiotics (Augmentin 625 mg, Ilsung Pharm. Co., Seoul, Korea) three times a day for 7 days. On clinical images taken at 2 weeks postinsertion, plaque bacteria were observed adhering to the healing abutment, but not the silicon cap, with only slight discoloration (Figure 3(a)). The cap also did not create a foreign body reaction or cause pain to the patient. Although the surrounding gingival margin was temporally suppressed by compression due to the silicon cap, healthy soft tissue regenerated, completely sealing the extraction socket (Figure 3(b)). At 2 months after the removal of the cap, the suppressed soft tissue had healed completely (Figure 3(c)). A final prosthesis was provided at 6 months after implant insertion, and healthy attached gingiva formed around the final prosthesis (Figure 3(d)), showing integration with the surrounding gingival margin, including the mucogingival junction (MGJ) line (Figure 3(e)). Furthermore, regenerated bone tissue was observed on the radiograph (Figure 3(f)). A radiograph taken at 22 months postoperatively also showed a well-maintained alveolar bone height (Figure 3(g)).

### 2.3. Case 2

**Figure 4:**
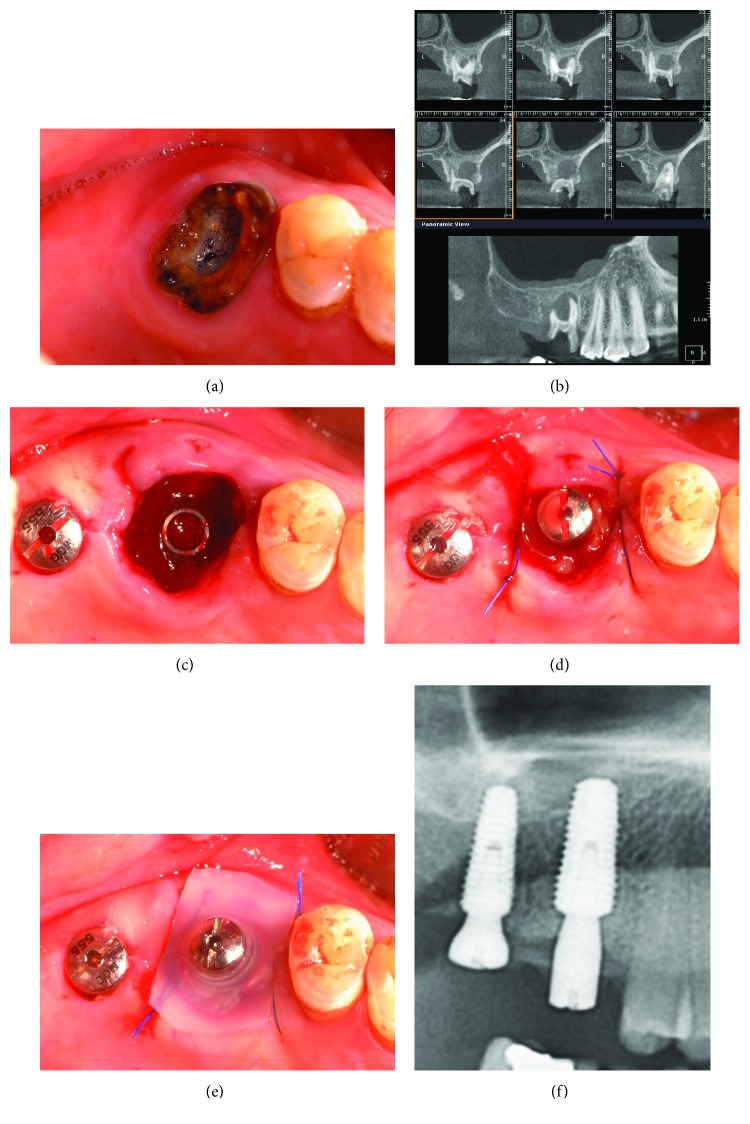
(a) Occlusal view at the initial examination. (b) Cone beam computerized tomography image at the initial examination. (c) Occlusal view immediately after the insertion of the implant. (d) Clinical condition of the interrupted sutures after gap filling of the extraction socket with allogenic bone and PRF. (e) Application of the silicone cap. (f) Radiograph taken immediately after implant insertion.

A 42-year-old male patient was admitted for a retained dental root and root apex lesion at tooth 16 and required an implant prosthesis for an extracted tooth 17 (Figure 4(a)). Computed tomography (Figure 4(b)) revealed that the retained bone height was insufficient due to the large root apex lesion of tooth 16. After atraumatic extraction of tooth 16, bone-added osteotome sinus floor elevation (BAOSFE) was performed using allogenic bone (SureOss, Osstem Implant Co., Seoul, Korea) to enhance the primary fixation of the implant. This BAOSFE procedure was performed using a drill and osteotome according to Summers' original technique [15]. A 13 mm long and 6 mm diameter implant (AnyOne, Megagen Implant Co., Seoul, Korea) was then immediately inserted with insertion torque exceeding 40 N cm (Figure 4(c)); the implant was inserted 1 mm deeper than the height of the alveolar ridge of the extraction socket. After collecting 10 cm^3^ of peripheral blood, platelet-rich fibrin (PRF) was prepared using a centrifuge (MF550, Hanil Science Industrial Co., Incheon, Korea). After application of the cylinder-type healing abutment, the gap in the extraction socket was filled with allograft material (SureOss, Osstem Implant Co., Seoul, Korea) up to the height of the alveolar ridge of the extraction socket, followed by filling with the PRF membrane up to the height of the soft tissue in the extraction socket (Figure 4(d)). Due to gingival tearing during extraction, two interrupted nonabsorbable sutures (Dafilon 5-0, B. Braun, Melsungen, Germany) were inserted. After fabricating a silicone cap as described earlier, it was placed over the healing abutment (Figures 4(e) and 4(f)). Using a flapless technique, a nonsubmerged 13 mm long and 5 mm diameter implant (AnyOne, Megagen Implant Co., Seoul, Korea) was inserted into the region of tooth 17, where the ridge had healed, accompanied by BAOSFE to enhance the primary fixation of the implant.

**Figure 5:**
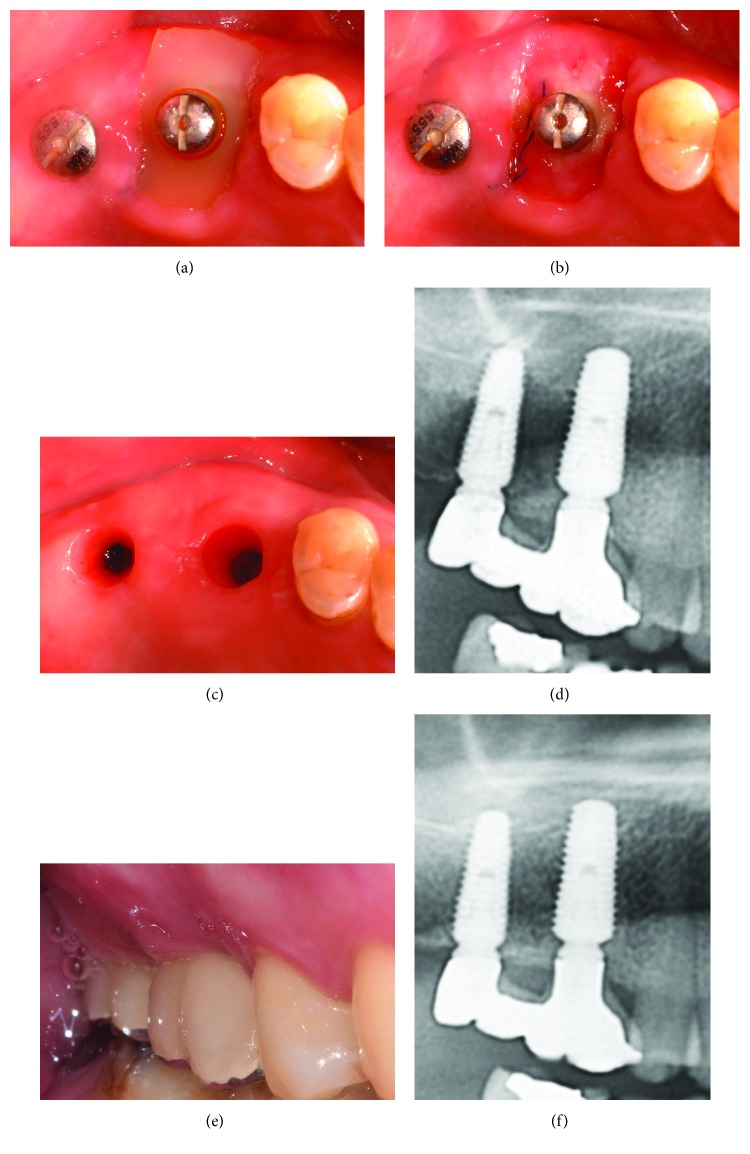
(a) Clean silicone cap at 2 weeks postoperatively. (b) Healing condition of the extraction socket at 2 weeks postoperatively. (c) Clinical condition before the application of the final prosthesis at 8 months after surgery. (d) Radiograph taken after the application of the final prosthesis at 8 months after surgery. (e) Buccal view at 2 years after surgery. (f) Radiographic view at 2 years after surgery.

At 2 weeks postinsertion, the cleanly maintained silicone cap and sutures were removed (Figure 5(a)); the extraction socket showed satisfactory healing, with soft tissue formation (Figure 5(b)). The final prosthesis was applied 8 months postoperatively. The soft tissue initially suppressed by the silicone cap was restored, and a well-formed attached gingiva was observed (Figure 5(c)). In addition, stable bone tissue was observed around the inserted implant neck, and increased vertical alveolar bone was observed under the implant (Figure 5(d)). Clinical and radiographic evaluation at 2 years postoperatively revealed a healthy gingival margin and satisfactory stability (Figures 5(e) and 5(f)).

## 3. Discussion

These cases demonstrate the potential for the application of a silicone cap in the immediate placement of molar implants. A silicone sheet is durable, flexible, thin, and easily manipulated and can completely cover the extraction socket or surgical area. Thus, it can prevent infection and protect the blood clot that is required for soft tissue healing at the extraction socket [13]. This can simplify the surgery required for the immediate placement of a dental implant, because the need for incision, dissection, periosteal-releasing incision, and suturing of the flap is eliminated or minimized. Additionally, since a coronally advanced flap is not required for extraction socket closure, the physiological MGJ line is preserved prior to extraction, enhancing the attachment of the gingiva.

Many previous attempts at sealing extraction sockets after immediate molar implant placement have been reported. Zafiropoulos et al. reported the use of a nonresorbable high-density polytetrafluoroethylene covering membrane in an open membrane technique [16]. Cafiero et al. reported that immediate transmucosal implant placement using a resorbable collagen membrane was a predictable treatment option [17]. However, both methods require flap elevation and complex sutures to place each barrier membrane precisely. Additionally, in the case of a nonresorbable membrane, additional invasive surgery is required to remove the barrier membrane. Jiansheng et al. attempted to seal the molar extraction socket by using a healing abutment with a wide diameter [18]. In this study, one of the 162 implants failed before prosthetic restoration, resulting in a success rate of 99.4%. However, this also required additional flap manipulation and sutures to attach the flap closely to the healing abutment, which caused some loss of gingival attachment. For full-mouth implant-supported rehabilitation of the upper jaw, Malchiodi et al. inserted immediately loaded implants after tooth extraction [19]. In this case, they attempted to attain extraction socket sealing using a temporary prosthesis. However, the indication for an immediately loaded implant is limited in the molar region, unlike in the anterior region. Thus, silicone caps may be an alternative to immediate molar implant placement.

**Figure 6:**
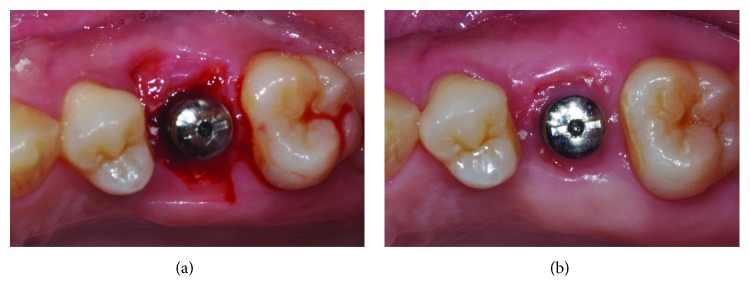
Other case that PRF membrane was not used for immediate implant placement in fresh maxillary molar extraction socket (tooth 26). (a) Poor healing condition of the extraction socket at 2 weeks postoperatively. (b) Healing condition of the extraction socket at 8 weeks postoperatively.

There are several points requiring attention in immediate implant placement using a silicone cap. The use of a PRF membrane is recommended for the extraction socket of both maxillary and mandibular molars when performing immediate implant placement using a silicone cap. For a mandibular molar, desirable healing of the soft tissue is observed only when a clot is present in the extraction socket at 2 weeks postoperatively, even without using PRF membrane under the silicone cap according to our clinical experience. For this reason, we generally recommend the removal of silicone caps at 2 weeks postoperatively. However, when the PRF membrane was not used for maxillary molars, poor healing was observed when the upper part of the extraction socket was filled with a mixture of new soft tissue and graft material used for gap filling (Figure 6(a)). Furthermore, it took 4-8 weeks longer for appropriate soft tissue healing in the extraction socket (Figure 6(b)). It has been postulated that this poor healing was caused by inhibition of soft tissue healing, because graft material from the maxilla was moved to the upper region of the extraction socket by gravity. In contrast, when a PRF membrane was used to cover the graft material, these issues were resolved (Figure 5(b)). In particular, the PRF membrane produced more favorable results due to the healing-promoting effect of growth factors on soft tissue in the extraction socket [20].

Moreover, it has been recommended that a cylinder-type healing abutment, rather than the tapered type, should be used. The silicone cap achieves sealing of the extraction socket due to the generated elasticity when a small punched hole is inserted over the healing abutment, requiring it to stretch as mentioned before (Figure 1(c)). However, it is impossible to obtain a complete seal of the extraction socket if the healing abutment is of the tapered type, where the diameter becomes smaller in the downward direction, and there is a chance that the silicone cap will be pushed inside the extraction socket, inhibiting its healing. In contrast, when using a healing abutment of the cylinder type, with the same diameter in its upper and lower parts, a stable extraction socket sealing effect is maintained over a period of time according to our clinical experience.

## 4. Conclusion

In these case reports, we have presented a new method for immediate implant placement after molar extraction using a silicone cap. Although it has been suggested that a good clinical outcome can be obtained with this technique, further investigation with long-term data and a larger sample size is needed to verify this approach.
